# The molecular control of GnRH neuron development

**DOI:** 10.1186/2193-1801-4-S1-L46

**Published:** 2015-06-12

**Authors:** Anna Cariboni, Andre´ Valentina, Kathryn Davidson, John Parnavelas

**Affiliations:** University of Milan, Italy; UCL, UK

**Keywords:** GnRH neuron, semaphorin, migration

Fertility critically depends by a small number of hypothalamic neurons secreting the neurohormone GnRH. During development GnRH neurons migrate from the nasal placode to the hypothalamus by following the migratory path formed by the vomeronasal axons. Developmental defects of this process can cause congenital GnRH deficiency (GD), characterized by absent/delayed puberty and consequent infertility. The underlying mutated loci are for the majority of GD cases unknown, partially because of a poor understanding of the molecular mechanisms that control the development of these crucial neuroendocrine cells. Here we provide evidence of the importance of class 3 semaphorins and their receptors in this process. Specifically, I will explain how two different semaphorins play distinct roles during the migration of GnRH neurons. Thus, semaphorin3A affects the migration of GnRH neurons in the nasal compartment, via the co-receptors Neuropilin-1 and 2, whereas semaphorin3E via its receptor plexind1 controls the survival of GnRH neurons once positioned in the hypothalamus. Accordingly, disrupted semaphorin signalling may be involved in the aethiopathogenesis of genetic diseases characterized by GnRH deficiency.
